# Establishing research impact assessment in Iran: The first report from a non-high-income country

**DOI:** 10.7189/jogh.14.04050

**Published:** 2024-03-15

**Authors:** Bahareh Yazdizadeh, Ayat Ahmadi, Farid Najafi, Kazem Mohammad, Mohammad Fariden, Davood Khalili, Mahdi Mahdavi, Elaheh Rahimpour, Abolghasem Jouyban, Roya Kelishadi, Mohammad Reza Monazzam, Monir Baradaran Eftekhari, Katayoun Falahat, Sima Nikooee, Reza Majdzadeh

**Affiliations:** 1 Knowledge Utilization Research Center, Tehran University of Medical Sciences, Tehran, Iran; 2 Research Center for Environmental Determinants of Health, Health Institute, Kermanshah University of Medical Sciences, Kermanshah, Iran; 3 Department of Epidemiology and Biostatistics, School of Public Health, Tehran University of Medical Sciences, Tehran, Iran; 4 Environmental Health Research Center, Department of Occupational Health and Safety at Work Engineering, Lorestan University of Medical Sciences, Khorramabad, Iran; 5 Prevention of Metabolic Disorders Research Center, Research Institute for Endocrine Sciences, Shahid Beheshti University of Medical Sciences, Tehran, Iran; 6 National Institute for Health Research, Tehran University of Medical Sciences, Tehran, Iran; 7 Pharmaceutical Analysis Research Center and Faculty of Pharmacy, Tabriz University of Medical Sciences, Tabriz, Iran; 8 Pharmaceutical Analysis Research Center and Faculty of Pharmacy, Tabriz University of Medical Sciences, Tabriz, Iran; 9 Child Growth and Development Research Center, Research Institute for Primordial Prevention of Non-Communicable Disease, Isfahan University of Medical Sciences, Isfahan, Iran; 10 Department of Occupational Health Engineering, School of Public Health, Tehran University of Medical Sciences; 11 Deputy for Research and Technology, Ministry of Health and Medical Education, Tehran, Iran; 12 Deputy for Research and Technology, Ministry of Health and Medical Education, Tehran, Iran; 13 School of Health and Social Care, University of Essex, Colchester, UK; * Equal contribution.

## Abstract

**Background:**

This study presents the first report on research impact assessment (RIA) in non-high-income countries, undertaken as a pilot initiative in 2021. Within it, we aimed to explore the feasibility of employing the ‘payback’ model for evaluating the impact of health research and enhancing the accountability of universities. We focussed on three key impact domains: ‘production of decision support documents and knowledge-based products,’ ‘implementation of research results,’ and ‘health and economic impact.’

**Methods:**

We adopted a case study approach to assess the impact of 5334 health research projects conducted by researchers from 18 universities from 2018 to 2020. Researchers were required to submit evidence related to at least one of the specified impact domains; six scientific committees verified and scored claimed impacts at the national level.

**Results:**

Only 25% of the assessed projects achieved impact in at least one domain, with the production of decision support documents and knowledge products being the most reported impact. Notably, economic impact was verified in only three projects, indicating room for improvement in this area. Technology research exhibited the highest acceptance rate of claimed impact, suggesting a positive correlation between technology-focused projects and impactful outcomes.

**Conclusions:**

This study demonstrates the feasibility of employing a case study approach and the ‘payback’ model to evaluate the impact of health research, even within the constraints of a moderately equipped research infrastructure. These findings underscore the potential of integrating RIA into the governance of health research in Iran and other non-high-income countries, as well as the importance of using RIA to assess the accountability of health research systems, guide the allocation of research funding, and advocate for the advancement of health research. The study sets a precedent for future assessments in similar contexts and contributes to the ongoing global dialogue on the societal impact of health research.

The significance of establishing and strengthening a national research system to meet the demands of a health system was emphasised at the 2008 Global Ministerial Meeting on Research for Health, held in Bamako, Mali [1]. In recent years, the role of research evidence in response to the coronavirus disease 2019 (COVID-19) pandemic has highlighted the value of research and the expectations of research systems [2]. All the initiatives emphasise the aim of research to make an impact on society, which should be promoted and investigated. In line with this, the World Health Organization (WHO) has outlined the importance of monitoring and evaluating in strengthening health research systems [3]. Furthermore, appropriate monitoring and evaluation are considered as one of the operational components of a health research system's stewardship in effectively managing its planning, implementation, and accountability [3,4].

Generally, the objectives of research impact assessment (RIA) are advocacy, accountability, analysis, and resource allocation [5]. Advocacy relates to preparing evidence to support funders’ decisions; accountability to showing that public funds are distributed correctly; analysis to identifying the best mechanism for funding and managing research; and resource allocation to prioritising the distribution of funds between individuals, projects, and organisations. By considering these two issues – the vision of health research system and the necessity of its monitoring and evaluation – it is essential to put the measuring of health research accountability at the heart of research impact assessment.

Accountability of research systems has not been clearly defined in the literature. For example, the ‘Guide to research evaluation frameworks and tools’ [6] describes it as showing ‘that money and other resources have been used efficiently and effectively, and to hold researchers to account.’ Meanwhile, the One Word Trust more comprehensively defines it as ‘the processes through which an organization makes a commitment to respond to and balance the needs of stakeholders in its decision-making processes and activities and delivers against this commitment’ [7].

In Iran, the integration of health and medicine into medical research and education in 1985 led to the establishment of the Ministry of Health and Medical Education (MOHME), with the purpose of increasing educational capacity and reducing shortages in the health workforce. This effort also aimed to promote synergism and bilateral accountability between higher education in the health and research system and the national health system [8,9]. The MOHME is responsible for the stewardship of health research in the country, while other research areas, such as engineering and social sciences, fall under the jurisdiction of the Ministry of Science, Research and Technology.

Currently, 69 medical universities in Iran fall under the purview of the MOHME, which is responsible for health, curative affairs, higher education, and research in various provinces, and whose Deputy for Research and Technology oversees the research governance for medical universities. The government allocates the research budget centrally to medical universities, which then administer grants to their researchers. Other granting bodies, such as the National Institute for Medical Research Development and the Iran National Science Foundation, also support health research projects in the country. Although the government funds most health research in Iran, when assessing the impact of medical universities’ research, we refer to any research conducted by academic staffs at medical universities, regardless of funding source.

Considering the increasing number of peer-review publications from Iran in recent years [8,9], the question of how beneficial the research output has been for the country is becoming increasingly relevant. Although the budget for research grew from 0.55% of gross domestic product (GDP) in 2001 to 0.87% in 2009, it fell short of the goal of 2.5% for 2015, with one of the reasons being a lack of belief among the country’s policymakers in the actual impact of research compared to other investments [10]. We therefore piloted this accountability assessment study for the first time in 2021, extending beyond academic impact, under the purview of the public research governance for research conducted in medical universities.

## METHODS

### Study arrangement

**Figure 1:**
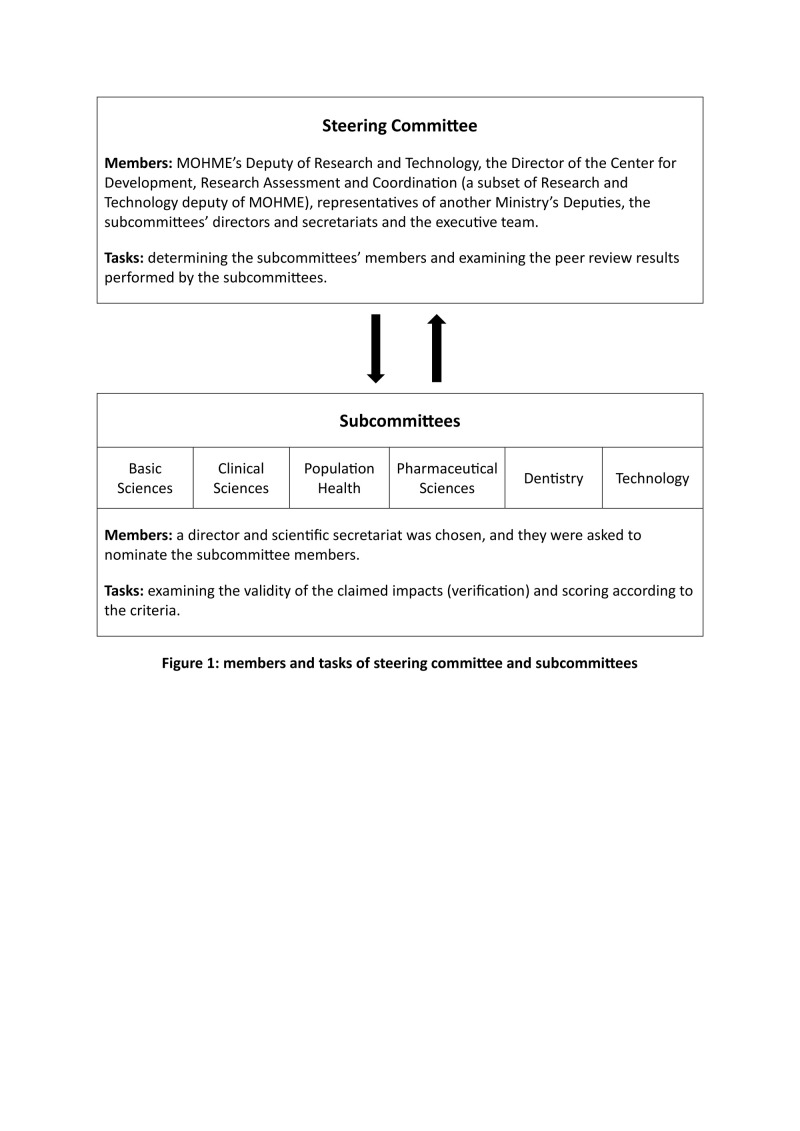
Members and tasks of steering committee and subcommittees.

We conducted this RIA using a case study approach in Iran’s medical universities. First, we established a steering committee with the responsibility to engage stakeholders, producers, and users of health research in all stages, including the design, data collection, analysis, and interpretation of the result. To this end, we invited well-known researchers and formal representatives from stakeholders, including different deputies in the MOHME responsible for various aspects of health (e.g. public health and primary care, curative affairs, and education) who are the potential users of research results in the country. After the initial meeting with the steering committee, given the challenges that have existed in various previous assessments, we jointly outlined and approved a set of principles, including the promoting approach (the assessment aims to strengthen the health research system to achieve its ultimate goals), equity (the participation of all researchers and universities and the encouragement/facilitation of all types of projects), transparency (all assessment procedures must be available to all stakeholders), and the control of conflict of interests (measures must be taken to prevent personal and organisational benefits in identifying and valuing research projects). We then created six sub-committees to review the stated impacts: basic sciences, clinical sciences, population health, pharmaceutical sciences, dentistry sciences, and technology (**Figure 1**).

We opted for a case study design for the RIA, as it examines research projects conducted over time to identify the changes they brought. This approach controls the attribution problem to some extent, but also has a considerable administrative burden [11]. Given that we sought to assess the accountability of medical universities in research and to use our findings to inform the Deputy for Research and Technology at the Ministry of Health and Medical Education for resource allocation, it was essential to attribute the impact of the research.

### Model and framework

To investigate effectiveness and efficiency of health research system, we first need to define and measure impact of research. In this assessment, we used the ‘payback’ logic model, which has been used more frequently than other models [12,13] and the pathway of research to impact and essential interfaces have been illustrated in the model comprehensively. The original ‘payback’ logic model from 1996 categorised the impact of research into five domains, including ‘knowledge production,’ ‘research targeting, capacity and absorption,’ ‘informing policies and product development,’ ‘health and health sector benefits,’ and ‘broader economic benefits’ [14]. Subsequent assessments have adapted critical elements of the ‘payback’ framework to make it more appropriate for a case study approach.

**Figure 2:**
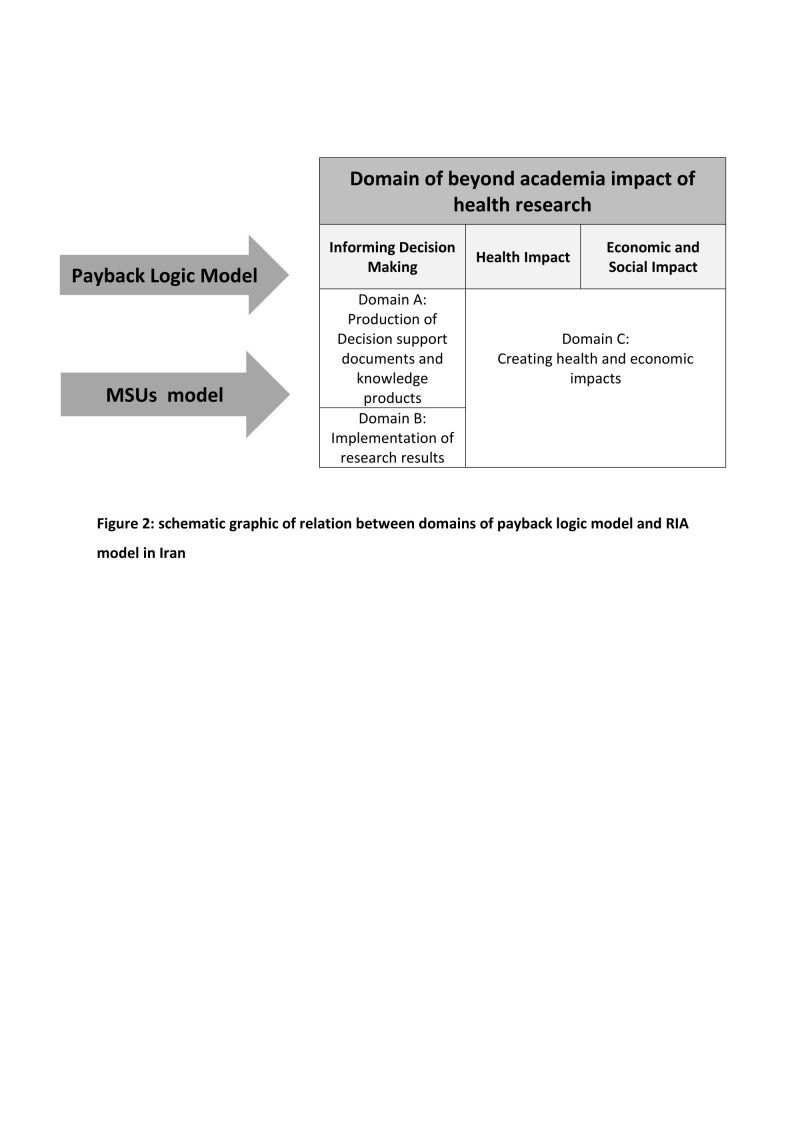
Schematic graphic of the relation between domains of payback logic model and RIA model in Iran.

To achieve the impact of research on health, society, and economics, the original ‘payback logic model contained a mediator stage defined as ‘informing policies and product development.’ This domain was changed to ‘informing decision making’ in the Canadian Institute of Health Research (CIHR) framework in 2005 [15] and to ‘policy impact’ and ‘health sector benefit’ in another study which evaluated existing approaches to cancer RIA in 2021 [12]. In our assessment, we split ‘informing decision making’ into two separate sub-domains: ‘production of decision support documents and knowledge-based products’ and ‘implementation of research results’ (**Figure 2**). Therefore, we have defined health research impact in three following domains.

#### Domain A: Production of decision support documents and knowledge products

We examined three indicators in this domain: Research conducted by request of an organisation outside universities (client-oriented research) (A-1); the production of decision support documentation and knowledge products (clinical guideline and public health guidance, Health Technology Assessment (HTA), patient decision support or policy brief) (A-2); and their implementation (A-3). Moreover, we considered two criteria for their assessment – the relevancy of the content and methodological quality.

#### Domain B: Implementation of research results

In this domain, we examined the utilisation of research results in the health systems decision-making by target groups. This domain could be reached from or independent of the pathway of domain A (**Figure 2**).

#### Domain C: Creating health and economic impacts

We also explored the health and economic impacts created by research projects. Health impact refers to the influence on disease occurrence, quality of life, or longevity achievable through effective interventions. It encompasses changes in people’s or patients' behaviour, improved case management, the identification of high-risk groups, modifications of health determinants (such as modifiable risk factors, social, and environmental determinants), and interventions that enhance the quality of health services, including aspects like acceptability, access, appropriateness, effectiveness, efficiency, and safety. Economic impact can result from various pathways, including the production and commercialisation of products or services (such as diagnostics, biologics, and personal or collective prevention measures), optimisation of existing products (improving quality or reducing marginal costs), knowledge-based entrepreneurship, reduction in the number of days lost due to disability, and the lowering of direct costs borne by patients and the health care system.

To verify and score this section, the utilisation of research results had to be approved by research users (domain B), and then the impacts created needed to be measured. Therefore, we could have only assessed this domain if the criterion for the second domain (domain B) was met.

Based on these three domains and their indicators, we developed an assessment form and then prepared a guide to help respond to the questions in its quality insurance section (Appendix 1 in the **Online Supplementary Document**).

### Sampling and data collection

We aimed to investigate the accountability of medical universities in Iran. As this was the first time that this assessment was to be conducted, we selected only a few medical universities to identify the practical challenges of doing such RIA in national level, as it was impossible to include all of them. Therefore, based on resources for assessment, we enrolled the top 18 medical universities (annually routine ranking based on academic impact). We asked researchers from these universities to complete relevant the form and upload it with the required approval documents in an online system. We included research projects whose impact was occurring in 2018–2020 (without considering the starting and finishing time of research). To corroborate the claimed impact [16], we clearly described the required approval documents in the guideline, which also emphasised that the researchers had to provide more than a letter from the research users claiming their utilisation of research results, as well as that the way of utilising research results and the decisions that have been affected by them should be clearly stated.

### Weighting and scoring

It is acknowledged that the selected domains will not have equal values in RIA. Thus, using the policy Delphi method [17], we used the stakeholders' opinions to assign weights to the domains. We sent the table of domains to the steering committee members, who were asked to give percent weights to all three domains (A, B, and C). Then, during a session with the steering committee, we discussed the reasons that caused outliers, after which we asked them to assign weights once again (**Table 1**). The ‘creating health and economic impacts’ domain has acquired the highest weight, although it did not differ significantly from the ‘implementation of research results’ domain.

**Table 1:** Weight scores for health research impact domains

Impact domain	Domain scope	Weight
Domain A	Production of decision aid documents and knowledge products: A1: Sponsor-initiated research projects (0.2); A2: Production of decision-aid documents and knowledge products (0.5); A3: Utilisation of the research result in decision aid documents and knowledge products (0.3)	0.25
Domain B	Implementation of research results	0.35
Domain C	Health and economic impacts of research projects	0.40

In determining the impact scores in each domain, we considered the impact's magnitude and its difficulty. These scores were determined by sub-committees and confirmed by steering committee members. The most determinant variable in assigning scores was the level of the impact (international, national, provincial, or district and hospital level), with modifying variables that changed the scores gained from the impact level (**Table 2**). We did not develop scoring criteria for health impact, as none of the claimed impact was found in this domain to be discussed further by committee members. Notably, the most peripheral level (district and hospital level) was given the most weight to encourage medical universities to conduct research for their population. Meanwhile, a minimum score was considered for investigating health and economic impact (by ignoring the impact happening or not).

**Table 2:** Method of scoring health research impacts

Impact domain	Level 1 indicator	Scoring criteria
Domain A: production of decision aid documents and knowledge products	A1: conducting sponsor-initiated research	The main determinant variable is the scope of sponsor's activities: international level: 5; national level: 4; provincial level (Academic): 3; district and hospital level: 2. Modified variable – collaborative sponsorship: two national clients: 5; if collaboration occurs at two levels one score will be added to the highest level. Budget provided by the main client: if the sponsor provides less than 25% of the project’s budget: 1 score will be downgraded; if the client provides more than 50% of the research budget: 1 will be upgraded. The amount of budget: if the total research budget exceeds 50 million Tomans: one score is added; if the total research budget is less than 20 million Tomans one score is reduced.
	A2: production of decision aid documents	The main determinant variable is the level of the approving organisation: international level: 5; national level: 4; provincial level (Academic): 3; district and hospital level: 2. If there was no approving organisation and only an article exists, it will score according to the scope of the document: international level: 5; national level: 4; provincial level (Academic): 3; district and hospital level: 2. Modified variable: if the project has a published article, one score will be added.
	A3: contribution of the results of research projects to decision-aid products	Included in systematic reviews: 1 score. Modulating variable: if the relevant systematic review has a registered protocol (e.g. in PROSPERO): 1.5 scores will be added; registered protocol in a peer review registration platform (such as Cochrane, or Campbell, JBI): 2 scores will be added. Regarding the utilisation of research in other documents, the score obtained depends on the application level of the relevant decision aid document: international level: 5; national level: 4; provincial level (Academic): 3; district and hospital level: 2.
Domain B: implementation of research results		In the field of knowledge, programs and policies – the main determinant variable is the level of implementation: international level: 5; national level: 4; provincial level (Academic): 3; district and hospital level: 2. Modified variable – the level of collaboration: if the collaboration of more than two organisations is necessary to implement the results, 1 score will be added. The relation of the research results and claimed impact: if they are acceptable another score will be added and if not, a score will be reduced. The score won't change in case of intermediate states. In the field of technological innovation and inventions – the main determinant variable is the level of the technology: super-advanced technology: 5 scores; advanced technology: 4 scores; average technology: 3 scores; basic technology (ordinary): 2 scores. Modified variable: deployment in a growth center/science and technology park due to the technological product resulting from the research project: 0.5 score will be added. Contract and memorandum with an accelerator through the technological product resulting from research: 0.5 score will be added. Establishing a knowledge-based company related to the technological product or idea (related to and based on the production and commercialisation of the product) resulting from the research: 0.5 score will be added. The license to produce or operate the product (license acquisition from the Health Ministry, license acquisition from the Medical University, license acquisition from the General Standard Office): 0.5 score will be added.
Domain C: impact on health and economy	Basic technology (ordinary): 2 score	In the technology and economic impacts domain, the main determinant variable is the level of the technology: super-advanced technology: 5 scores; advanced technology: 4 scores; average technology: 3 scores. Modified variable: internal sales; for every 200 million Iranian Rials 2 scores will be added; external sales (export); for every 1000 US dollars 2 scores will be added; the technological project’s implementation has resulted in employment and entrepreneurship; 5 scores are added for the annual employment of each individual.

### Quality assurance

To ensure the quality of collected data, we developed guidelines for various levels of users, including researchers and subcommittees. These guidelines defined the concepts within each domain, provided a list of required documents, and described methods for weighting and scoring. Additionally, we created an educational podcast and uploaded it to the assessment website, and organised educational sessions for university assessment experts and national-level researchers, while certain universities also conducted educational webinars for researchers.

### Analysis

Our approach to investigating accountability in research systems was done by joining the definition of accountability with the ‘3e’ approach, which encompasses effectiveness (does the research produce any outputs), efficiency (costs per unit of impact), and equity (does the research achieve health needs) [18]. Here we used effectiveness and efficiency to analyse accountability.

### Effectiveness

We presented the descriptive analyses of final scores for each medical university and each domain within each university. To enable comparison within and between universities, we standardised scores across domains A1, A2, and A3 within and between universities by dividing the mean score by the standard deviation.

### Efficiency

To calculate efficiency, we used effectiveness as the numerator and investment in research as the denominator. Since a significant proportion of research projects were student dissertations, and since faculty members’ salaries were also paid from the universities’ education budget, we used the universities’ overall research and education budget as an estimate of the budget spent on research. To compare universities, we set the highest score equal to 100 and calculated the scores of other universities based on that.

Participation in the assessment: We used the proportion of research projects submitted by each university (claimed impact) divided by the number of articles published in 2019 as a proxy index for university participation in the assessment. We assumed that all research projects would lead to a published article. Thus, the number of published articles was a proxy for the number of research projects at each medical university.

## RESULTS

Overall, 5334 research projects were forwarded to MOHME for final assessment. The universities' participation rates ranged from 2.6% to 55.0% (**Table 3**). Most submissions came from clinical research (n = 2054 (38.5%)), followed by basic sciences (n = 1458 (27.3%)) and population health research (n = 1265 (23.7%)). There were 524 (9.8%) submissions in technology, 416 (7.8%) in pharmaceutical sciences, and 398 (7.5%) in dentistry.

**Table 3:** Proportion of submitted projects of each medical university to the number of its publications in 2019*

University label	Number of publications in 2019†	Number of submitted projects	Proportion of submitted projects of each university to the number of publications in 2019
A	5915	353	5.97
B	4204	199	4.73
C	2782	90	3.24
D	1905	562	29.50
E	2129	821	38.56
F	2176	922	42.37
G	2204	623	28.27
H	1096	492	44.89
I	973	290	29.80
J	1245	154	12.37
K	882	485	54.99
L	858	59	6.88
M	817	33	4.04
N	376	91	24.20
O	233	56	24.03
P	496	72	14.52
Q	228	6	2.63
R	624	26	4.17
Total	29 143	5334	18.30

*This table shows the number of submitted projects by each participating university divided by the number of publications that the university had in 2019. This measure is considered an indicator of the university's participation rate.

†Publications indexed in ISI, PubMed, and Scopus.

Regarding effectiveness, 1341 (25.1%) research projects achieved at least one of the impact domains between 2018 and 2020. Decision support documents and knowledge products (domain A) were produced in 1327 research projects. Meanwhile, 110 research projects had been conducted based on sponsor needs and financial support (A1). Most of the scores were obtained from research results in systematic reviews. The research results were implemented (domain B) in 47 cases of research in 10 medical universities; most of this impact had been achieved by research product commercialization (**Box 1**). No research reported on health impact, while economic impact was verified in three research projects in one university.

The highest proportion of acceptance of claimed impact was observed in technology (222, 42.4%), followed by clinical research (668, 32.5%) and dentistry research (109, 27.4%).

Regarding efficiency, we observed that the rank of medical universities has changed. However, two medical universities, E and F, had nearly similar rankings in effectiveness and efficiency, which is a valuable characteristic of a university (**Table 4**, **Table 5**). The most frequently confirmed impact cases have been achieved in domain A (**Table 4**).

**Table 4:** Description of total score in each impact domain

Impact domain*	Total score	Impact score per university	Impact score per submitted project	Impact score per scored projects
Domain A	282.41	15.69	0.053	0.210
*A1*	23.1	1.28	0.004	0.017
*A2*	145.5	8.08	0.027	0.108
*A3*	113.81	6.32	0.021	0.085
Domain B	53.2	2.96	0.010	0.040
Domain C	3.6	0.20	0.001	0.003
Total	340.61	18.92	0.064	0.254
Adjusted for total research budget in 2019	174.79	9.71	0.033	0.130

*Domain A: Production of decision aid documents and knowledge products (A1: Sponsor-initiated research projects, A2: Production of decision-aid documents, A3: Contribution of the results of research projects to decision-aid products). Domain B: Implementation of research results. Domain C: Health and economic impacts of research projects.

**Table 5:** Crude and adjusted scores for each university in each domain

		Effectiveness/impact score†								Efficiency	
University label*	**Number of submitted projects**	**Domain A**				**Domain B**	**Domain C**	**Total score**	**University order according to effectiveness**	**Adjusted score‡**	**University order according to efficiency**
		**A1**	**A2**	**A3**	**Total A**						
E	242	1.90	13.00	24.71	39.61	9.45	0	49.06	1	17.25	3
F	113	0.30	25.13	7.43	32.85	12.07	3.6	48.5	2	19.73	1
A	97	7.20	22.63	3.34	33.16	9.8	0	42.96	3	8.86	9
G	218	2.00	12.13	27.04	41.16	0	0	41.16	4	18.07	2
D	149	3.90	19.50	10.05	33.45	6.47	0	39.9	5	16.47	4
H	211	1.80	7.50	18.23	27.53	4.2	0	31.72	6	18.06	2
K	101	0.15	12.63	8.40	21.18	0	0	21.18	7	16.27	5
J	21	0.25	7.75	0.90	8.90	4.2	0	13.1	8	13.87	6
I	24	1.60	7.88	1.20	10.68	0	0	10.68	9	9.80	8
P	40	0.30	1.38	3.94	5.61	3.32	0	8.94	10	12.51	7
B	30	0.85	3.88	3.08	7.80	0	0	7.8	11	1.86	15
C	16	1.35	4.38	0.15	5.88	1.05	0	6.92	12	2.58	14
N	41	0.00	0.50	3.94	4.44	1.57	0	6.015	13	7.84	10
L	17	1.30	2.75	0.30	4.35	0	0	4.35	14	3.07	13
O	13	0.20	3.38	0.53	4.10	0	0	4.1	15	5.47	11
Q	3	0.00	0.00	0.26	0.26	1.05	0	1.31	16	3.09	12
R	5	0.00	0.63	0.34	0.96	0	0	0.96	17	-	-
M	2	0.00	0.50	0.00	0.50	0	0	0.5	18	-	-
Total	1343	23.10	145.50	113.81	282.41	53.20	3.6	340.61		174.79	

*Universities are ordered based on their total impact score and labelled based on their number of faculty members.

†Domain A: Production of decision aid documents and knowledge products (A1: Sponsor-initiated research projects, A2: Production of decision-aid documents, A3: Contribution of the results of research projects to decision-aid products). Domain B: Implementation of research results. Domain C: Health and economic impacts of research projects.

‡Adjusted for total research budget in 2019.

**Figure 3:**
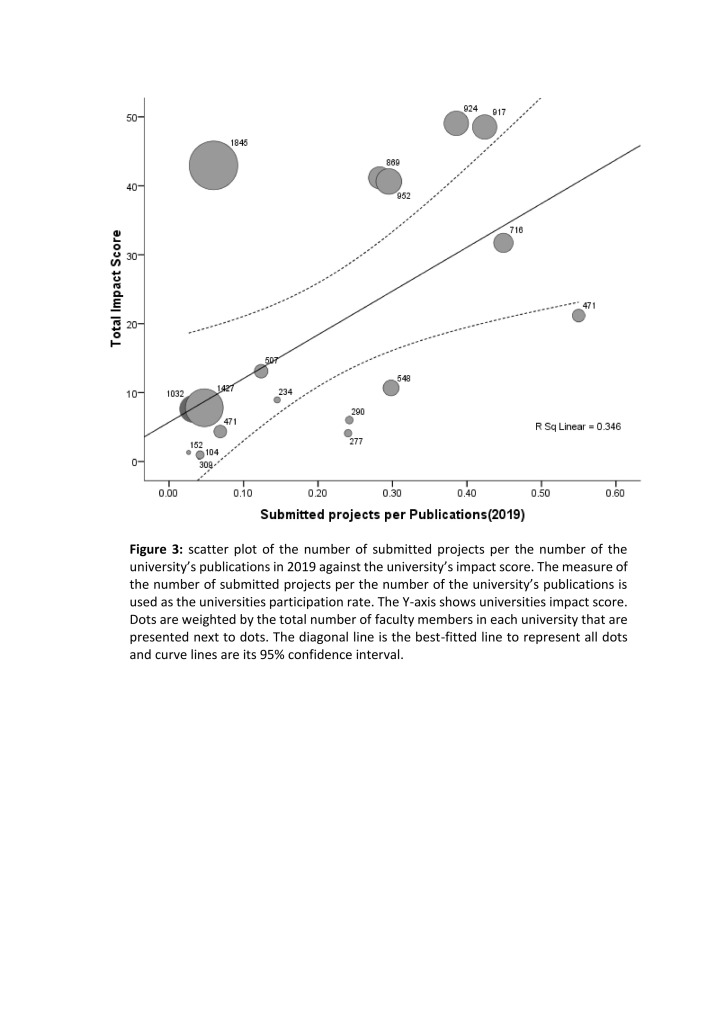
Scatter plot of the number of submitted projects per the number of the university’s publications in 2019 against the university’s impact score.

There was a positive linear correlation (r = 0.59) between the participation rate and the impact score for universities. Larger universities had lower participation rates in the assessment (**Figure 3**).

## DISCUSSION

We conducted this study across medical universities to assess the feasibility of conducting RIA and using its results to investigate their accountability.

### Application of RIA

Although there is limited global evidence on using RIA results for decision-making, there are two notable examples. The first is the Program Assessment Rating Tool (PART) used by the United States Office of Management and Budget to assess and improve the performance of Federal programmes. Based on its result, programmes are classified as effective (if the score is between 85 and 100), moderately effective (if it is between 70 to 84), adequate (if it is between 50 and 69), and ineffective (if the score is less than 49) [19]. The second is the Research Excellence Framework (REF) in the UK, which uses the proportion of world-leading and internationally excellent submitted research studies to decide on funding allocation [20].

Our assessment confirmed that measuring the accountability of the health research system based on two elements, effectiveness and efficiency, is possible. As previously mentioned, we merged the definition of accountability with the ‘3e’ approach, which was essential to investigate accountability to use the result in decisions related to resource allocation and advocacy.

The RIA’s results should be understood with several factors in mind. Although domain A achieved the highest score, this should be interpreted cautiously. Providing the document for confirmation of the claimed impact in domain B was difficult because we had asked the researcher to prepare a document with the signature of the results’ end-user, which had to clearly explain how the research results had been implemented. Furthermore, to receive a score in domain C, it was necessary to confirm the impact in domain B. This was challenging for both researchers and users. However, in research projects with a technology orientation, it was easier to prepare confirmation documents (e.g. patent certificate and amount of product sales); moreover, we observed a higher technology orientation impact rather than a health impact in domain C. Considering this difference in preparing confirmation documents, this result is not far from our expectations. Previous research in Iran found sub-optimal utilisation of research results in the decision-making of the health system [21-23].

### Methodological significance

To investigate the effectiveness and efficiency of the health research system, we need to define and measure the impact of research. Some frameworks for assessing the impact of research are similar in domain and indicators of academic impact. Still, they differ in their assessment objectives, indicators to investigate beyond academic impact, data collection methods, and utilisation of results. For example, in 2009, the Canadian Academy of Health Science (CAHS) defined health research impact domains as ‘knowledge advancement,’ ‘capacity building,’ ‘informing decision-making,’ ‘health,’ and ‘socioeconomic impacts’ [24]. Based on the request of the Deputy of Research and Technology of the MOHME and the steering committee decision, and considering that the first two domains have been examined in the assessments started in 2001, we selected the last three domains of the 1996 ‘payback’ logic model for assessment, as (knowledge advancement domain is the core component of research system evaluation at the individual and organisational levels and capacity building in terms of career promotion and the number of the thesis are some parts of educational system evaluation) [18].

This study had several unique methodological characteristics. First, we defined new constructs, specifically for the ‘informing decision-making’ domain in the ‘payback’ model. These constructs include ‘producing decision aid documents and knowledge products’ and the ‘implementation of research results,’ assuming that the latter could occur independently of the former. The need for such constructs was previously identified in empirical research, such as one on the RIA of 46 cancer studies which found that 36 of them had recommendations for policymakers [25]. This issue is also addressed in a new guide published by the WHO, which includes a domain of ‘evidence production,’ such as health technology assessment reports, policy briefs, and clinical guidelines (known as third-party research) as the output of research can affect decision-making [26]. Second, in contrast to many RIAs that prioritise international and national impacts, we focussed on the impact of research on the local community. This emphasis on local impacts encourages universities to be responsive and accountable to the needs of the communities in which they operate. Third, we employed a structured tool for data collection and open-ended questions to ensure comprehensibility for respondents. This approach differs from the narrative approach used in some RIAs, such as the REF in the UK, which requests researchers to describe the impacts of their research in the form of storylines. Lastly, to address the challenge of stakeholders having different interpretations of the term ‘impact,’ we provided clear definitions for the term in each domain to ensure consistency in the assessment of research impacts.

#### Remedying challenges

In this study, we adhered to eight out of ten recommendations for conducting health RIA [19], as we did not observe two recommendations: ‘Stakeholder participation’ and a ‘mixed-methods approach.’ We did not involve patients and the public in the stakeholder participation, while the complete participation of some policymakers and managers from other MOHME departments was not feasible, despite our efforts. Although we checked the validity of the researchers' statements during the analysis stage, we did not adopt a mixed-methods approach during the data collection stage. However, we developed the impact scoring method with the participation of a wide range of experts and stakeholders.

However, we encountered a significant challenge in the low participation of universities and researchers in the assessment program. Different levels of participation by universities made the scores less comparable, creating a significant challenge in interpreting the findings. The low participation of researchers could be attributed to two main factors: The challenging circumstances surrounding COVID-19 and the lack of individual motivation for researchers. To increase researchers' participation in future assessments, the assessment results of universities should be linked to those of researchers' activities. Despite some universities' low participation, some of them obtained high scores (**Figure 3**). Increased researcher participation is expected to raise the attained score. However, some universities with high participation rates did not achieve high scores due to the reasons previously described. Therefore, it is expected that increasing researchers' familiarity with this subject will increase their participation rate in future assessments.

We propose several recommendations to address the challenges observed in this assessment and improve the validity of the assessment results. The first is to increase researchers' involvement in the assessment program by linking the university's assessment to the researchers' assessment. This will incentivise researchers to undertake knowledge translation activities and participate more in the assessment program. The second is to remove the committees of pharmaceutical sciences, dental sciences, and technology and consider three domains: Basic sciences, clinical sciences, and population health. Specialists from the former three sciences should be included in these three latter committees to optimize the review process. The third recommendation is to create new databases and use routinely available data from different units of MOHME.

### Further research

To enhance the validity and reliability of health RIA and increase its utilisation in improving the health research system, we propose several directions for further research. First, a comparative analysis of the impacts generated by different science domains, such as basic, clinical, and population health, could be performed to identify their inherent differences in creating impact, the suitability of the assessment framework, and the role of the peer review process in the achieved results. Second, the mechanisms of creating effect need to be examined to identify the intervention points that lead to increased research effectiveness and efficiency. Through these studies, the utilisation of RIA in the decision-making process of the health system can be optimised, and the overall accountability of the health research system can be improved.

## CONCLUSIONS

All health research impact assessments have traditionally been conducted in high-income countries with well-established data systems and infrastructures. However, funding mechanisms for health research vary greatly in non-high-income countries. Therefore, we aimed to investigate the feasibility of using a case study approach to assess the impact of health research in a setting with limited routine data infrastructure. Despite the challenges, the results demonstrate that RIA can be conducted in such settings and can be generalized to other low- and middle-income countries. Furthermore, our findings highlight the importance of utilising RIA results for decision-making, particularly in assessing the accountability of the health research system. The results can inform research financial allocation and advocacy efforts for strengthening health research systems.

Box 1 **Examples of research that its impact in domains A2 and B (other than the commercialisation of products).**

- ‘Training and implementation of urinary tract infection prevention package on the rate of urinary tract infection related to an indwelling urinary catheter in patients hospitalized in special care units.’
- ‘Reviewing the teaching method of learning professional medical ethics using two methods of role-playing and rethinking.’
- ‘Internal controls of health centres' cash in medical university.’
- ‘Designing evidence-based clinical guidelines for nursing care in children with thalassemia major.’
- ‘Development and implementation of ethical guidelines in palliative care for terminal patients.’

## Additional material


Online Supplementary Document
