# Supplementary material for: Establishing research impact assessment in Iran: The first report from a non-high-income country
Source: J Glob Health. 2024 Mar 15;14:04050. doi: 10.7189/jogh.14.04050 (PMC10939117; doi:10.7189/jogh.14.04050)
Supplement: Online Supplementary Document [file jogh-14-04050-s001.pdf]

Questionnaire code:

Questionnaire of the project "Evaluation of the effects of research conducted in universities of medical sciences"

Greetings and Regards

The ultimate goal of research is to improve the health and well-being of communities. To achieve this goal, research should be done based on the needs and its results should be used in the decisions of health stakeholders. In order to promote the use of health research results in health-related decisions, the first step is to measure it. For this reason, the evaluation of the performance of the country's medical sciences universities based on the index of research effects has been prioritized in the programs of the vice president of research and technology, so that, in addition to drawing the current situation, points that need intervention can be identified and effective interventions implemented.

In order to evaluate the effect of health research, frameworks and indicators have been proposed at the international level, the most important of which have been used in this evaluation are the Production of decision support documents and knowledge products, implementation of research results, and creating health and economic impacts.

Therefore, you, respected researchers, are requested to review the questions that have been raised in the following form and if your researches during the years 2017, 2018 and 2019 caused at least one of the effects stated in the form, have, please complete the form and send it along with the required documents to the research and technology vice-chancellor of the university.

The areas of the intended effects and the required documentation are stated in the "Protocol of the evaluation program of medical sciences universities of the Islamic Republic of Iran based on the indicators of health research effects of the researcher's version".

Note:

The year of the approval of the research and the year of its termination are not important, the important thing is that the impact of the research took place in the three years determined.

A separate evaluation form should be completed for each effective study.

Thanks

Questionnaire code:

Research Characteristics:	
Project title:	
University:	
Code of ethics:	Project code in the university research system:
Place of proposal approval (university/research center):	
Main researcher:	Title of organizational affiliation :

Part A: Production of Decision support documents and knowledge products

1- Has the research been done on the order of the decision-making organization?

Yes Name of ordering organization: ....

No

2- Has the research led to the production of one of the following decision-making documents?

 Preparation of educational content for policy makers and managers, service providers, service recipients, students, public
 Preparation of service delivery guide including Patient Decision Aid, clinical practice guideline and public health guidance
 Preparation of Health Technology Assessment
 Preparation of Policy Brief
 Preparation of policy documents, instructions or approvals of implementing organizations
 Domestic patent registration
 International patent registration

3- Have the research results been used in the compilation of studies and documents to help the following decisions?

 Educational content for policy makers and managers, service providers, service recipients, students, public
 Regular review studies (including all types of review studies)
 Service delivery guide including Patient Decision Aid, clinical practice guideline and public health guidance
 Health Technology Assessment
 Policy Brief
 Policy documents, instructions or approvals of implementing organizations

Questionnaire code:

Part B: Implementation of research results

4- Have the results of this project been used in the decisions of the Ministry of Health (directly or indirectly)?

 Yes No/I don't know

If your answer is yes, please explain how to use:
....
....
....

5- Have the results of the mentioned plan been used in decisions outside the health system (directly or indirectly)?

 Yes No/I don't know

If your answer is yes, please explain how to use:
....
....
....

6- Have the results of this plan been used in the decision-making of the province, university, or the health and treatment network of the city, in your place of service?

 Yes No/I don't know

If your answer is yes, please explain how to use:
....
....
....

Part C: Creating health and economic impacts

7- Does the application of the results of this project have health effects, including the impact on the prevalence and incidence of disease, quality of life, or longevity?

 Yes No

The effect on health can be created from the following paths, please specify in which way the research results have led to the creation of health effects:

 Conducting effective interventions at the community level to prevent disease
 Effect on people's behavior and effect on patients' behavior
 Cheaper, faster, more accurate diagnosis / determination of immunogenicity and safety diagnosis
 More appropriate management of hospitalized patients
 Using effective treatment or support methods
 Identification of high-risk groups for contracting the disease

 Conducting interventions affecting the status of health determinants (such as modifiable risk factors, social determinants and environmental determinants)
 Carrying out interventions affecting the quality of health services (including acceptability, access, appropriateness, effectiveness, efficiency and safety)
 Other cases:..

8- Has the application of the results of this project led to economic effects? Yes No

The effect on the economy can be created from the following paths, please specify in which way the research results have led to the creation of economic effects:

 Production of new marketable products or services (such as the production of diagnostic methods, vaccines, personal or collective prevention equipment)
 Optimizing the previous product or product (increasing quality or reducing production cost)
 Knowledge-based entrepreneurship
 Reducing the number of days lost from work due to illness or disability (admission at home or hospital)
 Reducing the direct costs of patients
 Reducing the direct costs of the health system
 Other items:...

9- Dear researcher, please write about the effects of your research and what you have chosen in the previous questions. If you need more explanations, please write in this section. These explanations will help in the judging process.

....
....
....

Steering Committee

Members: MOHME’s Deputy of Research and Technology, the Director of the Center for Development, Research Assessment and Coordination (a subset of Research and Technology deputy of MOHME), representatives of another Ministry’s Deputies, the subcommittees’ directors and secretariats and the executive team.

Tasks: determining the subcommittees’ members and examining the peer review results performed by the subcommittees.

Subcommittees

Basic Sciences	Clinical Sciences	Population Health	Pharmaceutical Sciences	Dentistry	Technology

Members: a director and scientific secretariat was chosen, and they were asked to nominate the subcommittee members.

Tasks: examining the validity of the claimed impacts (verification) and scoring according to the criteria.

Figure S1. Members and tasks of steering committee and subcommittees.

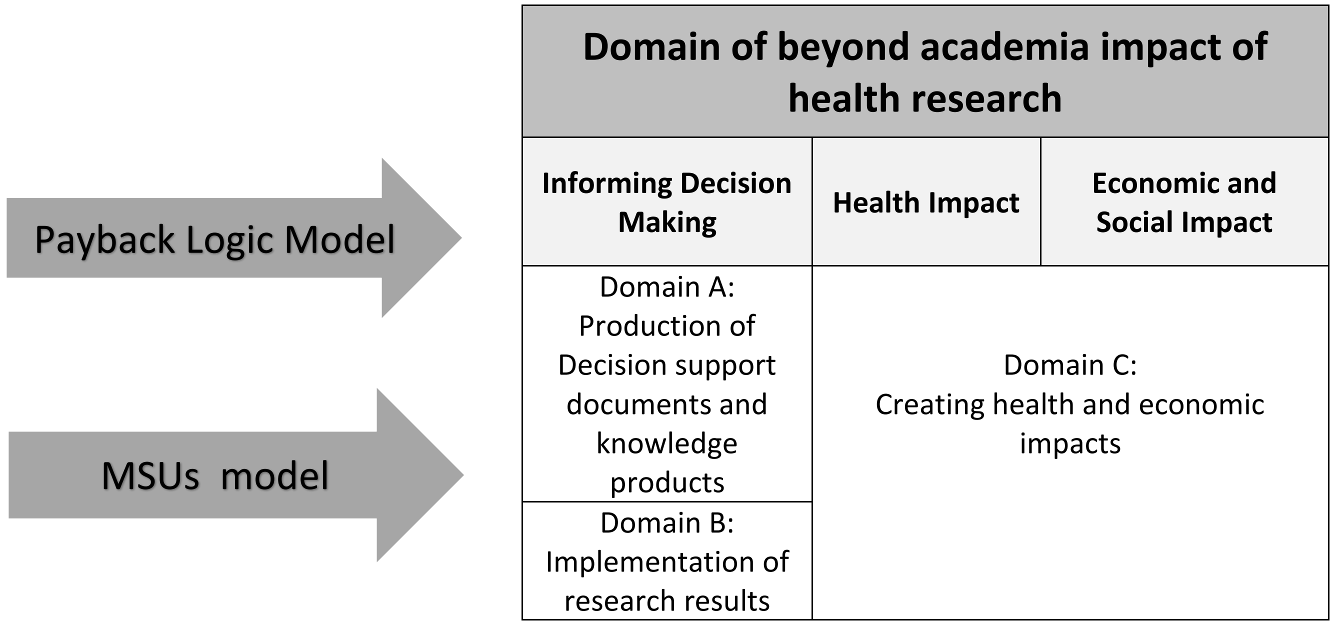

Figure S2. Schematic graphic of relation between domains of payback logic model and RIA model in Iran.

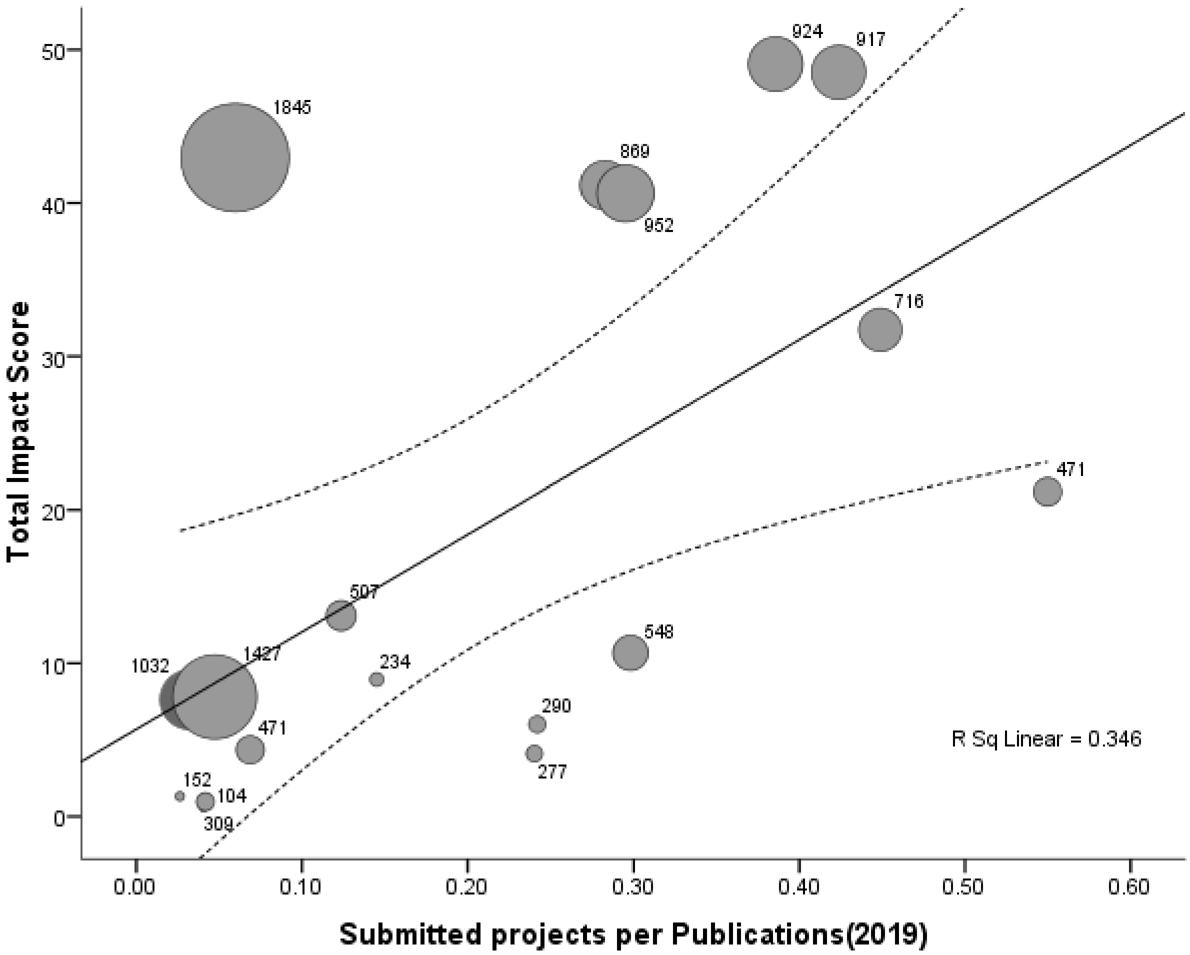

Figure S3. Scatter plot of the number of submitted projects per the number of the university's publications in 2019 against the university's impact score. The measure of the number of submitted projects per the number of the university's publications is used as the universities participation rate. The Y-axis shows universities impact score. Dots are weighted by the total number of faculty members in each university that are presented next to dots. The diagonal line is the best-fitted line to represent all dots and curve lines are its 95% confidence interval.

Table 1. Weight scores for health research impact domains

Impact domain	Domain scope	weight
Domain A	Production of decision aid documents and knowledge products:<br>A1: sponsor-initiated research projects (0.2)<br>A2: production of decision-aid documents and knowledge products (0.5)<br>A3: utilization of the research result in decision aid documents and knowledge products (0.3)	0.25
Domain B	Implementation of research results	0.35
Domain C	Health and economic impacts of research projects	0.40

Table 2. Method of scoring health research impacts

Impact domain	Level 1 indicator	Scoring criteria
**Domain A: Production of decision aid documents and knowledge products**	A 1: Conducting sponsor-initiated research	The main determinant variable is the scope of sponsor's activities:<br>International level: 5<br>National level: 4<br>Provincial level (Academic): 3<br>District and hospital level: 2<br><br>Modified variable:<br>Collaborative sponsorship:<br>Two national clients: 5<br>If collaboration occurs at two levels one score will be added to the highest level.<br><br>budget provided by the main client:<br>If the sponsor provides less than 25% of the project's budget: 1 score will be downgraded<br>If the client provides more than 50% of the research budget: 1 will be upgraded<br><br>the amount of budget:<br>If the total research budget exceeds 50 million Tomans: one score is added.<br>If the total research budget is less than 20 million Tomans one score is reduced.
	A 2: Production of decision aid documents	The main determinant variable is the level of the approving organization:<br>International level: 5<br>National level: 4<br>Provincial level (Academic): 3<br>District and hospital level: 2<br>If there was no approving organization and only an article exists, it will score according to the scope of the document:<br>International level: 5<br>National level: 4<br>Provincial level (Academic): 3<br>District and hospital level: 2<br><br>Modified variable:

		If the project has a published article, one score will be added.
	A 3: contribution of the results of research projects to decision-aid products	Included in systematic reviews: 1 score<br>Modulating variable:<br>If the relevant systematic review has a registered protocol (e.g. in PROSPERO): 1.5 scores will be added<br>registered protocol in a peer review registration platform (such as Cochrane, or Campbell, JBI): 2 scores will be added<br>Regarding the utilization of research in other documents, the score obtained depends on the application level of the relevant decision aid document:<br>International level: 5<br>National level: 4<br>Provincial level (Academic): 3<br>District and hospital level: 2
**Domain B: Implementation of research results**		**In the field of knowledge, programs and policies:**<br>The main determinant variable is the level of implementation:<br>International level: 5<br>National level: 4<br>Provincial level (Academic): 3<br>District and hospital level: 2<br>Modified variable:<br>The level of collaboration:<br>if the collaboration of more than two organizations is necessary to implement the results, 1 score will be added.<br>The relation of the research results and claimed impact:<br>if they are acceptable another score will be added and if not, a score will be reduced. The score won't change in case of intermediate states.<br><br>**In the field of technological innovation and inventions:**<br>The main determinant variable is the level of the technology:<br>Super-advanced technology: 5 scores<br>Advanced technology: 4 scores<br>Average technology: 3 scores<br>Basic technology (ordinary): 2 scores

		Modified variable::<br>Deployment in a growth center/science and technology park due to the technological product resulting from the research project: 0.5 score will be added.<br>Contract and memorandum with an accelerator through the technological product resulting from research: 0.5 score will be added.<br>Establishing a knowledge-based company related to the technological product or idea (related to and based on the production and commercialization of the product) resulting from the research: 0.5 score will be added<br>The license to produce or operate the product (license acquisition from the Health Ministry, license acquisition from the Medical University, license acquisition from the General Standard Office….): 0.5 score will be added
**Domain C:**<br>**Impact on health and economy**	Basic technology (ordinary): 2 scores	In the technology and economic impacts domain,<br>the main determinant variable is the level of the technology:<br>Super-advanced technology: 5 scores<br>Advanced technology: 4 scores<br>Average technology: 3 scores<br>✓ Modified variable:<br>Internal sales; for every 200 million Iranian Rials 2 scores will be added<br>External sales (export); for every 1000 US dollars 2 scores will be added<br>The technological project's implementation has resulted in employment and entrepreneurship; 5 scores are added for the annual employment of each individual

Table 3. Proportion of submitted projects of each medical university to the number of publications in 2019 by it

University label	Number of publications* in 2019	Number of submitted projects	Proportion of submitted projects of each university to the number of publications in 2019 (%)
A	5915	353	5.97
B	4204	199	4.73
C	2782	90	3.24
D	1905	562	29.50
E	2129	821	38.56
F	2176	922	42.37
G	2204	623	28.27
H	1096	492	44.89
I	973	290	29.80
J	1245	154	12.37
K	882	485	54.99
L	858	59	6.88
M	817	33	4.04
N	376	91	24.20
O	233	56	24.03
P	496	72	14.52
Q	228	6	2.63
R	624	26	4.17
Total	29143	5334	18.30

This table shows the number of submitted projects by each participating university divided by the number of publications that the university had in 2019. This measure is considered an indicator of the university's participation rate.

*ISI, PubMed and Scopus indexed publications.

Table 4. Description of total score in each impact domain

Impact domain*	Total score	Impact score per university	Impact score per submitted project	Impact score per scored projects
Domain A	282.41	15.69	0.053	0.210
A1	23.1	1.28	0.004	0.017
A2	145.5	8.08	0.027	0.108
A3	113.81	6.32	0.021	0.085
Domain B	53.2	2.96	0.010	0.040
Domain C	3.6	0.20	0.001	0.003
Total	340.61	18.92	0.064	0.254
Adjusted for total research budget in 2019	174.79	9.71	0.033	0.130

*Domain A: Production of decision aid documents and knowledge products (A1: sponsor-initiated research projects, A2: production of decision-aid documents, A3: contribution of the results of research projects to decision-aid products), domain B: implementation of research results and domain C: health and economic impacts of research projects.

Table 5. Crude and adjusted scores for each university in each domain

University label[a]	Number of submitted projects	effectiveness/Impact score[b]								Efficiency	
		Domain A				Domain B	Domain C	Total score[c]	University order according to effectiveness	Adjusted score [d]	University order according to efficiency
		A1	A2	A3	Total A						
E	242	1.90	13.00	24.71	39.61	9.45	0	49.06	1	17.25	3
F	113	0.30	25.13	7.43	32.85	12.07	3.6	48.5	2	19.73	1
A	97	7.20	22.63	3.34	33.16	9.8	0	42.96	3	8.86	9
G	218	2.00	12.13	27.04	41.16	0	0	41.16	4	18.07	2
D	149	3.90	19.50	10.05	33.45	6.47	0	39.9	5	16.47	4
H	211	1.80	7.50	18.23	27.53	4.2	0	31.72	6	18.06	2
K	101	0.15	12.63	8.40	21.18	0	0	21.18	7	16.27	5
J	21	0.25	7.75	0.90	8.90	4.2	0	13.1	8	13.87	6
I	24	1.60	7.88	1.20	10.68	0	0	10.68	9	9.80	8
P	40	0.30	1.38	3.94	5.61	3.32	0	8.94	10	12.51	7
B	30	0.85	3.88	3.08	7.80	0	0	7.8	11	1.86	15
C	16	1.35	4.38	0.15	5.88	1.05	0	6.92	12	2.58	14
N	41	0.00	0.50	3.94	4.44	1.57	0	6.015	13	7.84	10
L	17	1.30	2.75	0.30	4.35	0	0	4.35	14	3.07	13
O	13	0.20	3.38	0.53	4.10	0	0	4.1	15	5.47	11
Q	3	0.00	0.00	0.26	0.26	1.05	0	1.31	16	3.09	12
R	5	0.00	0.63	0.34	0.96	0	0	0.96	17	-	-
M	2	0.00	0.50	0.00	0.50	0	0	0.5	18	-	-
Total	1343	23.10	145.50	113.81	282.41	53.20	3.6	340.61		174.79	

[a]Universities are ordered based on their total impact score and labeled based on their number of faculty members.
[b]Domain A: Production of decision aid documents and knowledge products (A1: sponsor-initiated research projects, A2: production of decision-aid documents, A3: contribution of the results of research projects to decision-aid products), domain B: implementation of research results and domain C: health and economic impacts of research projects.
[d]Adjusted for total research budget in 2019
